# Tuberculous Pleurisy Diagnosed From Massive Pleural Effusion in an Older Patient With No History of Tuberculosis

**DOI:** 10.7759/cureus.32333

**Published:** 2022-12-08

**Authors:** Miki Nanyoshi, Shiho Amano, Taichi Fujimori, Chiaki Sano, Ryuichi Ohta

**Affiliations:** 1 Family Medicine, Shimane University Faculty of Medicine, Izumo, JPN; 2 Community Care, Unnan City Hospital, Unnan, JPN; 3 Internal Medicine, Shimane University, Izumo, JPN; 4 Community Medicine Management, Shimane University Faculty of Medicine, Izumo, JPN

**Keywords:** frailty, massive pleural effusion, tuberculous pleurisy, rural hospital, general medicine

## Abstract

Tuberculous pleurisy is an infectious disease with a poor prognosis needing early diagnosis. The use of appropriate antituberculosis drugs can improve prognosis. However, the diagnosis of tuberculous pleurisy is often challenging in older patients. Decreased activities of daily living (ADLs) may lead to difficulty in performing invasive procedures to make a definite diagnosis of pleural effusion. We report our experience with a 90-year-old female with the chief complaint of dyspnea with massive pleural effusion. We could not perform an intensive investigation for tuberculous pleurisy. Based on the high value of adenosine deaminase (ADA), we tentatively diagnosed tuberculous pleurisy for the large pleural effusion and treated her well with the initiation of four antituberculosis drugs. ADA in pleural effusion is considered effective for diagnosis among dependent older patients. Furthermore, although it is difficult to diagnose tuberculous pleurisy in older patients, starting treatment to sustain older patients’ lives in their homes is crucial.

## Introduction

Massive pleural effusions are often caused by malignancies, parapneumonic effusions, tuberculosis, or exudative pleural effusions. Moreover, it has been reported that more than 90% of lymphocyte-dominant exudative pleural effusions are caused by tuberculosis and malignant diseases [1]. Tuberculous pleurisy is an infection of tuberculosis in pleural spaces and develops when the initial foci of infection that occur directly beneath the pleura spread to the pleura. The infection may also be concomitant with the development of active pulmonary tuberculous foci and continuous spread to the pleura or by hematogenous spraying [2]. The diagnosis of tuberculous pleurisy is defined as tuberculosis bacteria detected in sputum, pleural effusion, and pleural biopsy material, and histopathologically specific granulomas detected by pleural biopsy [2]. The standard treatment for tuberculous pleurisy is isoniazid (INH), rifampicin (RIF), ethambutol (EB), and pyrazinamide (PZA) for two months and INH and RIF for four months. These drugs have significant side effects, especially liver enzyme elevation, fever, skin rashes, and visual disturbances [3].

In an aging society, the incidence of tuberculous pleurisy in older adults is increasing, but diagnosis is often difficult. Invasive inspection is challenging owing to progressive frailty and low activities of daily living (ADLs). Thus, it may be hard to obtain evidence for diagnosis [4]. We report our experience with a 90-year-old female from a nursing home with a chief complaint of dyspnea. We subsequently found a large left-sided pleural effusion. The possible investigations showed the results of the analysis of pleural effusion with lymphocyte dominance and high adenosine deaminase (ADA) levels. Tuberculous pleurisy was clinically diagnosed and treated with four antituberculosis medicines, leading to a cure of symptoms and discharge to the nursing home. Through this case, we discuss the difficulty in diagnosing tuberculous pleurisy in older patients and the importance of prompt treatments for tuberculous pleurisy for sustaining older patients’ quality of life.

## Case presentation

A 90-year-old female in a nursing home visited our hospital with the chief complaint of dyspnea. A day before the visit, she experienced stomach discomfort, and the oxygen saturation (SpO2) measured at the facility was in the 70% range without difficulty breathing. After deep breathing, the SpO2 became around 90%. Around noon on the day of the visit, she visited her family doctor because of dyspnea, and her SpO2 level was recorded at 60%-80%. Her family doctor referred her to our hospital for suspected pneumonia and pulmonary embolism. Her past medical history included hypertension, dementia, glaucoma, osteoporosis, osteoarthritis in both knees, and pseudogout of the right knee. A review of records showed that she was taking the following medications: donepezil, amlodipine, acetaminophen, lubiprostone, and sennoside.

**Figure 1:**
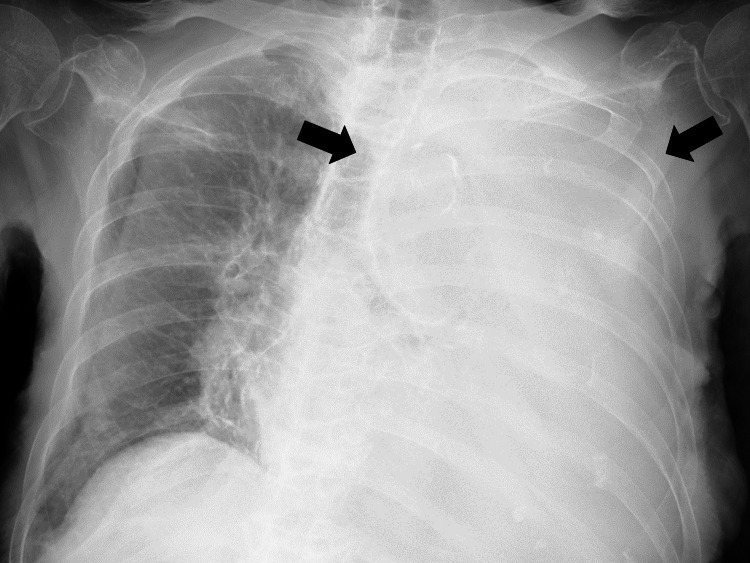
Chest X-ray showing left pleural effusion (black arrows)

Vital signs at the consultation were as follows: body temperature of 37°C, blood pressure of 115/71 mm Hg, pulse rate of 82 beats/minute, respiratory rate of 20 breaths/minute, and 88% SpO2 (10 L/minute, mask with reservoir). Her consciousness was alert. Physical findings showed that the conjunctiva of the eyelids was pale, with no head, neck, or axillary lymph node swelling. Respiratory sounds decreased on the left side of the lungs. Pitting edema was observed on both sides of the lower leg. The nail bed color was slightly pale, and the skin turgor was slightly decreased. Chest radiography revealed a large pleural effusion in the left lung (Figure 1).

Simple and contrast-enhanced computed tomography (CT) of the chest and pelvic cavity showed no obvious lymphadenopathy or malignancy without any abnormal shading of the lungs, except for massive left pleural effusion based on radiology consultation. She was admitted to the department of general medicine. The results of blood tests are shown in Table 1.

**Table 1:** Laboratory data of the patient (blood and pleural effusion) eGFR: estimated glomerular filtration rate; UIBC: unsaturated iron-binding capacity; TIBC: total iron-binding capacity; ESR: erythrocyte sedimentation rate; T-SPOT: T-cell spot test for tuberculosis; sIL-2R: soluble interleukin-2 receptor; PT: prothrombin time; INR: international normalized ratio; APTT: activated partial thromboplastin time; N: neutrocyte; L: lymphocyte; LDH: lactate dehydrogenase; CEA: carcinoembryonic antigen; CA19-9: carbohydrate antigen 19-9; ADA: adenosine deaminase

Marker	Level	Reference
White blood cells	4.3 × 10^3^/μL	3.5-9.1 × 10^3^/μL
Neutrophils	78.1%	44%-72%
Lymphocytes	10.5%	18%-59%
Monocytes	8.9%	0%-12%
Eosinophils	1.8%	0%-10%
Basophils	0.7%	0%-3%
Red blood cells	3.08 × 10^6^/μL	3.76-5.50 × 10^6^/μL
Reticulocyte	1.1%	0.4%-1.9%
Hemoglobin	10.2 g/dL	11.3-15.2 g/dL
Hematocrit	29.9%	33.4%-44.9%
Mean corpuscular volume	97 fl	79-100 fl
Platelets	31 × 10^4^/μL	13-36.9 × 10^4^/μL
Total protein	6.7 g/dL	6.5-8.3 g/dL
Albumin	2.2 g/dL	3.8-5.3 g/dL
Total bilirubin	0.3 mg/dL	0.2-1.2 mg/dL
Direct bilirubin	0.1 mg/dL	0-0.4 mg/dL
Aspartate aminotransferase	19 IU/L	8-38 IU/L
Alanine aminotransferase	6 IU/L	4-43 IU/L
γ-Glutamyl transpeptidase	6 IU/L	<48 IU/L
Lactate dehydrogenase	219 U/L	121-245 U/L
Uric acid	3 mg/dL	3-6.9 mg/dL
Blood urea nitrogen	8.81 mg/dL	8-20 mg/dL
Creatinine	0.61 mg/dL	0.40-1.10 mg/dL
eGFR	67.6 mL/minute/L	>60 mL/minute/L
Serum Na	133 mEq/L	135-150 mEq/L
Serum K	3.3 mEq/L	3.5-5.3 mEq/L
Serum Cl	101 mEq/L	98-110 mEq/L
Serum Ca	8.1 mg/dL	8.8-10.2 mg/dL
Serum Fe	24 μg/dL	48-154 μg/dL
Ferritin	29.5 ng/mL	14.4-303.7 ng/mL
UIBC	160 μg/dL	108-325 μg/dL
TIBC	184 μg/dL	246-410 μg/dL
Occult blood stool	Negative	Negative
Vitamin B12	870 pg/mL	180-914 pg/mL
Folic acid	<2.2 pg/mL	>4 pg/mL
ESR (one hour)	55 mm	2-15 mm
T-SPOT	Negative	Negative
sIL-2R	748 U/mmL	154-474 U/mmL
PT	107.3%	70%-130%
PT-INR	0.96	
APTT	32.2 seconds	25-40 seconds
Fibrinogen	375.6 mg/dL	200-400 mg/dL
D-dimer	8.2 μg/mL	～1 μg/mL
pH	7.567	
Color	Brownish yellow	
Specific gravity	1.030	
Germ	-	-
Differential white blood cell (Giemsa) N:L	3:97	
Red blood cell	>50/HPF	<1/HPF
White blood cell	>50/HPF	<1/HPF
Histiocyte	<1/HPF	<1/HPF
Mesothelial cell	<1/HPF	<1/HPF
Total protein	4.1 g/dL	g/dL
Albumin	1.55 g/dL	g/dL
LDH	123 IU/L	IU/L
Glucose	116 mg/dL	mg/dL
CEA	1.9 ng/mL	<10 ng/mL
CA19-9	<2.9 U/mL	<2.9 U/mL
ADA	85.2 U/L	<40 U/L

There was an increase in neutrophil fraction, erythrocyte sedimentation rate, and anemia. T-SPOT was negative, and the soluble interleukin-2 receptor level was slightly high. Thoracentesis was performed because of the massive left-sided pleural effusion, 1,400 mL of yellowish-brown pleural effusion was removed, and a chest drain was placed. Pleural effusion examination revealed exudative pleural effusion with lymphocyte dominance and high ADA levels (Table 1). Sputum cultures were obtained for three consecutive days starting on the fourth day of hospitalization, but the results were negative. The drainage was serous, blood stained only on the first day, and then serious, and the drainage gradually decreased. The total volume was approximately 2,800 mL over six days. No decrease in pleural effusion or worsening of the clinical course was noted, and the drainage catheter was removed.

The differential diagnosis was cancerous pleural effusion secondary to solid cancer, malignant lymphoma, or tuberculous pleurisy. Cancerous pleural effusion due to solid cancer was less likely than tuberculous pleurisy because there was no history of malignancy, the tumor markers were negative, and chest and abdominal CT scans showed no neoplastic lesions or enlarged lymph nodes. Clinically, we diagnosed tuberculous pleurisy based on the dominant cell growth of lymphocytes and high ADA values. However, sputum culture and T-SPOT were negative, and a pleural biopsy could not be performed because of the patient’s poor general condition. Based on high ADA elevation and the exclusion of other diseases, we clinically diagnosed her with tuberculous pleurisy and initiated a four-drug combination therapy of INH, RIP, EB, and PZA on the 14th day of hospitalization. Based on the test results, anemia associated with chronic inflammation and folic acid deficiency was diagnosed. Ferrous citrate and folic acid were administered to treat the anemia. Subsequently, she was discharged to the previous nursing home due to decreased oxygen demand, improved respiration, and better appetite, and continued antituberculosis therapy.

## Discussion

In this case, tuberculous pleurisy was suspected because of the high pleural effusion ADA level and lymphocyte-significant blood cytosis without negative tuberculosis bacteria future. Eventually, the patient with tuberculosis treatment could return to the previous living condition with the recovery of respiratory conditions. This case shows the difficulty of diagnosing tuberculous pleurisy and the importance of precise clinical reasoning and treatment based on the reasoning, even in older patients.

Clinical evaluation is important for the differential diagnosis of massive pleural effusion and tuberculous pleurisy when a pleural biopsy is impossible. Massive exudative pleural effusions are commonly caused by malignancies, parapneumonic pleural effusions, and tuberculosis [2]. Here, the pleural effusion satisfied the Light’s criteria (protein ratio: 4/6.7 > 0.5; LDH: 128 less than the upper limit of normal serum LDH (211 × 2/3 = 140)), and we judged it as exudative pleural effusion. Additionally, it was a lymphocyte-dominant (neutrophils/lymphocytes = 3:97) exudative pleural effusion. Although the causes of exudative pleural effusion are diverse, including tuberculosis, malignancies, pulmonary embolism, and malignancies, they account for more than 90% of all reported cases [1]. Therefore, we believe that it is essential to distinguish between pleurisy caused by malignancy and by tuberculosis.

Pleural effusion due to tuberculous pleurisy is suspected when there is early progression (within one week or one month) and yellowish-brown, lymphocyte-dominant pleural effusions. The specificity of the diagnosis of tuberculous pleural effusion was 80% if the percentage of lymphocytes in the pleural effusion was greater than 90% [3] and 90% if the ADA value was 80 or more. Here, both criteria were met, and the probability of tuberculous pleurisy was high. The diagnosis was defined as evidence of tuberculosis bacteria in sputum, pleural effusion, or pleural biopsy material, and histopathologically specific granulomas confirmed by pleural biopsy. However, both sputum cultures were negative in this case, and T-SPOT was negative. However, the positivity rate in sputum cultures is only 40% [4]. The sensitivity of T-SPOT is 81%-88%, and its specificity is more than 90% [5], but false-negative results of T-SPOT in active tuberculosis increase in patients aged 80 years and older (sensitivity of 81.6% below 80 years and sensitivity of 74.2% above 80 years) [5]. False-negative results may have been present in this case.

Malignant pleural effusion has high specificity for the presence or absence of a history of malignant tumors, dull chest pain, large pleural effusion, pleural effusion blood contamination, and abnormal shadowing of the chest radiograph; the possibility of malignant pleural effusion increases if these are present. On the other hand, if there is a fever of 37°C or higher and a period of illness within 30 days, the possibility is reduced. In addition, the presence or absence of an abnormal shadow on chest CT has high sensitivity and specificity [6,7]. Tumor markers of pleural fluid are also useful for diagnosis. The specificity of CEA (cutoff value: 5 ng/mL) was 98.6%. The specificity of CA19-9 (cut-off value: 100 ng/mL) is reported to be 100% [8], which is very high and can be used as a reference for definitive diagnosis [9]. On the other hand, the positivity rate of pleural effusion tumor markers is high, at approximately 70%, which cannot be used as a basis for excluding the diagnosis of malignant tumors. The sensitivity of pleural effusion cytology is as low as 75% [10].

The precise differential diagnosis based on clinical reasoning is essential for diagnosing tuberculous pleurisy, leading to effective treatment. In our case, malignant pleural effusion was initially differentiated, as there was no history of malignant tumors, serous drain drainage, no abnormal shadows on chest CT, and no malignant cells in pleural effusion. Therefore, the probability of a malignant tumor was considered low. However, there are no highly sensitive examinations other than chest CT regarding carcinomatous pleurisy. Therefore, pleural effusion cytology must be repeated to rule out carcinomatous pleurisy, even after starting treatment with antituberculosis drugs. In addition, a pleural biopsy is useful for diagnosing both malignant pleural effusion and tuberculous pleurisy, but a biopsy could not be performed because an invasive examination was impossible owing to the risk of complications among older patients [11].

While the methods of diagnosing tuberculous pleurisy from massive pleural effusion in older patients are limited, disease progression may significantly reduce activities of daily living (ADLs) in older patients [12]. In this case, effective treatments of tuberculous pleurisy can alleviate older patients’ conditions and increase the possibility of returning to previous living conditions. Especially in rural contexts, the population is aging, and more older patients come to hospitals with pleural effusions. In this article, general physicians have to deal with the issues comprehensively. As system-specific specialists, general physicians should perform effective clinical reasoning and differential diagnosis of pleural effusions, contributing to the effective treatment of tuberculous pleurisy [13]. Effective care of older patients can be achieved by excluding diagnoses and accelerating the clinical diagnosis and treatment of tuberculous pleurisy.

## Conclusions

Tuberculous pleurisy in older adults is challenging to diagnose clinically. Making an appropriate diagnosis for exclusion and clinical diagnosis is important. It is important to comprehensively assess the condition of older patients and initiate appropriate tuberculosis treatment to improve their general condition and maintain their quality of life in communities.
